# Effects of Microbial Inoculation and Storage Length on Fermentation Profile and Nutrient Composition of Whole-Plant Sorghum Silage of Different Varieties

**DOI:** 10.3389/fmicb.2021.660567

**Published:** 2021-04-13

**Authors:** E. Cole Diepersloot, Matheus R. Pupo, Lucas G. Ghizzi, Jessica O. Gusmão, Celso Heinzen, Cody L. McCary, Marcelo O. Wallau, Luiz F. Ferraretto

**Affiliations:** ^1^Department of Animal and Dairy Sciences, University of Wisconsin, Madison, WI, United States; ^2^Department of Animal Sciences, University of Florida, Gainesville, FL, United States; ^3^Department of Animal Nutrition and Animal Production, University of São Paulo, Pirassununga, Brazil; ^4^Department of Animal Science, Federal University of Lavras, Minas Gerais, Brazil; ^5^Agronomy Department, University of Florida, Gainesville, FL, United States

**Keywords:** *L. buchneri*, *L. diolivorans*, variety, aerobic stability, 1, 2-propanediol.

## Abstract

This study aimed to assess the effects of a heterofermentative microbial inoculant and storage length on fermentation profile, aerobic stability, and nutrient composition in whole-plant sorghum silage (WPSS) from different varieties. Experiment 1, a completely randomized design with a 2 × 3 factorial treatment arrangement, evaluated microbial inoculation [CON (50 mL distilled water) or LBLD (*Lactobacillus plantarum* DSM 21762, *L. buchneri* DSM 12856, and *L. diolivorans* DSM 32074; 300,000 CFU/g of fresh forage)] and storage length (14, 28, or 56 d) in forage WPSS. The LBLD silage had lower pH compared to CON, and greater concentrations of succinic acid, ethanol, 1,2-propanediol (1,2-PD), 1-propanol, 2,3-butanediol and total acids. After 56 d, lactic acid concentration was greater for CON, while acetic acid and aerobic stability were greater in LBLD silage. Experiment 2, a completely randomized design with a 2 × 3 factorial treatment arrangement, evaluated effects of microbial inoculation (same as experiment 1) and storage length (14, 28, or 56 d) in WPSS of three varieties [forage sorghum (Mojo Seed, OPAL, Hereford, TX), sorghum-sudangrass (Dyna-gro Seed, Fullgraze II, Loveland, CO, United States), or sweet sorghum (MAFES Foundation Seed Stocks, Dale, MS State, MS)]. The LBLD forage sorghum had greater acetic acid and 1,2-PD concentrations at 56 d and 28 d, respectively, but lower concentrations of propionic acid at 56 d and butyric acid at 14 and 28 d. Additionally, WSC concentration was greater for CON than LBLD at 28 d. Furthermore, CON sweet sorghum had greater lactic acid, propionic acid, and butyric acid concentrations. However, greater acetic acid and 1,2-PD were observed for LBLD sweet sorghum. The CON sweet sorghum had greater concentration of WSC and yeast counts. The CON sorghum sudangrass had greater lactic and butyric acid concentrations than LBLD at 14 d, but lower acetic acid and 1,2-PD concentrations at 56 d. Yeast counts were greater for CON than LBLD sorghum sudangrass silage. Overall, results indicate inoculation of WPSS with *Lactobacillus plantarum* DSM 21762, *L. buchneri* DSM 12856, and *L. diolivorans* DSM 32074 improves heterofermentative co-fermentation allowing the accumulation of acetic acid concentration and increasing antifungal capacities and aerobic stability of WPSS.

## Introduction

Sorghum production is gaining popularity with dairy producers in the United States, especially in regions that experience drought, delayed planting, and high summer temperatures which limit corn production (Dann et al., 2008; Hasan et al., 2017). Recently, studies have been focused on improving sorghum characteristics as a forage crop, including increasing yield and nutritive value (Reddy and Reddy, 2003; Kertikov, 2007). With a wide range of types and varieties available, it is of ultimate importance to select adapted materials that require low inputs, could recover from drought, have high yield potential under dryland regions, and high forage quality.

Commonly used sorghum types, such as sorghum-sudangrass and forage sorghum, are popular with growers because of their flexible planting time, rapid growth, high yields, suitability in rotation systems, and high nutritive value (McDonald et al., 1991; Cothren et al., 2000). Another line of breeding focused on developing sweet sorghum materials for bioenergy use because of its adaptability, high dry matter (DM) yield, growth characteristics (Knoll et al., 2018), and high concentration of fermentable sugars (Zhang et al., 2016). Although data embracing its use as silage is lacking, the chemical composition suggests it may be suitable for silage production, as water-soluble carbohydrates (WSC) are the primary substrate utilized by lactic acid bacteria (LAB) for growth at the beginning phase of ensiling, leading to a drastic reduction in pH (Yang et al., 2006), which is essential for silage preservation.

Microbial inoculants are a frequently used tool by dairy producers trying to influence silage fermentation and improve aerobic stability (Krooneman et al., 2002; Kleinschmit and Kung, 2006; Kung et al., 2018), but literature is controversial. Typically, traditional homofermentative microbial inoculants (mainly producing lactic acid) have been the preferred choice of producers in the United States to increase the rate of sorghum silage fermentation. Nevertheless, the second generation of heterofermentative microbial inoculants producing lactic and antifungal acids (i.e., acetic and propionic acids) from the degradation of other fermentation byproducts offer greater advantages. These contain a combination of strains from different species such as *Lactobacillus diolivorans* and *L. buchneri* (Kung et al., 2018), which can increase aerobic stability in sorghum silage. Inoculants containing a combination of both strains could enhance acetic and propionic acid production, which are antifungal compounds that suppress yeast and mold growth. The bacteria *L. buchneri* produces 1,2-propanediol (1,2-PD) during the degradation of lactic acid into acetic acid (Oude Elferink et al., 2001), and *L. diolivorans* can utilize 1,2-PD as a carbon source for growth, producing propionic acid and 1-propanol as the main fermentation end products (Zielińska et al., 2017). Capitalizing on a co-fermentation with both microbial strains can result in the production of more antifungal acids, improving aerobic stability and silage fermentation. Although the use of *L. buchneri* is well-established, its combination with new strains is receiving renewed attention (Fernandes et al., 2020; Ferrero et al., 2021).

Storage length is positively related to aerobic stability in silages inoculated with heterofermentative inoculants. Greater concentrations of acetic acid inhibit yeasts, resulting in enhanced aerobic stability (Kung et al., 2018), but the conversion of lactic acid into acetic acid was thought to begin after approximately 30 to 60 d of storage length (Driehuis et al., 1999; Muck et al., 2018). More recent studies demonstrated the onset of acetic acid production may begin as early as 15 d of storage (Fernandes et al., 2020), depending on bacterial strain and silo conditions. Besides, producers are often challenged with silage shortages, requiring silos to be opened before ideal circumstances would normally permit. In 2019, for example, abnormally wet spring conditions delayed corn planting for many producers, and for some prevented it altogether, leading to a record 11.4 million acres of corn unplanted in the U.S. (Schnepf, 2020). Conditions like these, and others such as limited growing area, create difficulties for producers without enough silage carryover from previous years. Delayed planting can encourage producers to turn to sorghum crops because of their more rapid maturation, which allows them to be harvested before corn.

Therefore, two experiments were conducted to assess the effects of adding heterofermentative microbial inoculants in whole-plant sorghum silage (WPSS). The objective of experiment 1 was to examine the effect of a microbial inoculant containing *L. plantarum* DSM 21762, *L. buchneri* DSM 12856, and *L. diolivorans* DSM 32074 and storage length on the fermentation profile, aerobic stability, and nutrient composition of WPSS. The objective of experiment 2 was to determine the effects of microbial inoculation with *L. plantarum* DSM 21762, *L. buchneri* DSM 12856, and *L. diolivorans* DSM 32074, and storage length on fermentation profile, nutrient composition and NDF ruminal disappearance of WPSS made from forage sorghum, sweet sorghum, and sorghum sudangrass. We hypothesized that microbial inoculation would improve the fermentation profile and aerobic stability of WPSS, and that this response would increase with storage length and be of greater magnitude in varieties with greater soluble sugar concentrations.

## Materials and Methods

### Experiment 1

This experiment was carried out in North Florida during the summer growing season of 2019. A completely randomized design with a 2 × 3 factorial arrangement of treatments was used to evaluate the effect of microbial inoculation [CON (50 mL distilled water) or LBLD (Provita Supplements Inc., Mendota Heights, MN, *Lactobacillus plantarum* DSM 21762, *L. buchneri* DSM 12856, and *L. diolivorans* DSM 32074; 300,000 CFU/g of wet forage)] and storage length (14, 28, or 56 d) in WPSS.

#### Crop Establishment, Harvesting, and Ensiling

Sorghum forage (Ascend BMR MS Sorghum; DFA Farm Supplies, Syracuse, NY) was obtained from a commercial dairy farm (Alliance Dairies, Trenton, FL, United States) grown during the summer season. Fertilizer, pesticide, and herbicide applications were applied according to protocols established by Alliance Dairies and harvest timing was based on the farm harvest schedule. Forage was harvested from four locations (used as replication; 4 mini-silos per treatment combination [microbial inoculant by storage length]) within the same field on October 29th, 2019 at approximately 22% DM with a self-propelled forage harvester (Claas of America, Omaha, NE, united States) using a theoretical length of cut of 17 mm, without a kernel processor and without internal inoculant application. To ensure no contamination from previous microbial inoculant residue in the harvester, approximately 3 m of forage was harvested and then discarded.

Microbial inoculation rates were based on counts determined by pour plating on Man, Rogosa, Sharpe agar (Oxoid, Blasingstoke, United Kingdom). Microbial inoculant was diluted in distilled water targeting 300,000 CFU/g wet forage of *Lactobacillus plantarum* DSM 21762, *L. buchneri* DSM 12856, and *L. diolivorans* DSM 32074 (Provita Supplements Inc., Mendota Heights, MN, United States), applied and hand-mixed with chopped forage immediately before ensiling. Those not treated with LBLD were treated with distilled water in the same proportion (CON). Forage was packed in laboratory scale silos (20 L plastic buckets) considered as experimental units at a density of approximately 180 kg of DM/m^3^. After packing, buckets were closed and sealed with tape to avoid aerobic exposure until reaching the targeted fermentation lengths (14, 28, or 56 d). Therefore, the experiment consisted of 6 treatments (two microbial inoculations × three storage lengths) and 24 silos (four replications per treatment combination).

#### Chemical Analysis

Fresh, uninoculated forage samples were collected for each of the four locations to establish a baseline for nutritive value, DM, pH and yeast, and mold counts. An undried and unground sample of 20 g was diluted 10-fold (mass basis) in 0.1% peptone water (Oxoid CM0090), blended for 60 s in a high-speed stomacher (Lab-Blender 400, Tekmar Company, Cincinnati, OH, United States), and filtered through two layers of cheese cloth to extract forage juice. Forage extract was collected, and pH was immediately measured in duplicate using a pH meter (Corning model 12, Corning Scientific Instruments, Medfield, MA, United States). In addition, a separate sample of forage extract was added to a 50 mL plastic tube to evaluate yeast and mold enumeration via a pour plating method in a 10-fold serial dilution on malt agar (Difco 211220) acidified with 85% lactic acid. Agar plates were incubated at 32°C for 48 h for yeast, and an additional 72 h for mold counts. Yeast and mold counts were evaluated on the same plates and separated by visually examining the growth of colonies. Fresh samples from each plot were dried in a forced-air oven set at 60°C for 48 h to determine DM content, and sufficiently dried to be ground to pass through a 1-mm sieve using a Wiley Mill (Thomas Scientific, Swedesboro, NJ) and sent to Rock River Laboratory Inc. (Watertown, WI) for chemical characterization. Absolute DM was determined by oven-drying at 105°C for 3 h (method 2.2.2.5; National Forage Testing Association, 1993). Samples were analyzed for DM, crude protein (CP; method 990.03; AOAC International, 2012), ether extract (EE; method 920.39; AOAC International, 2012), starch (Hall, 2015), water-soluble carbohydrates (WSC; Dubois et al., 1956), and ash (method 942.05, AOAC International, 2012). Neutral detergent fiber was determined using α-amylase and sodium sulfite (aNDF; method 2002.04, AOAC International, 2012).

After experimental silos reached their assigned storage length, buckets were opened, and the top 10 cm of material was discarded. Immediately after opening, the material was homogenized, and a sub-sample of approximately 250 g was collected and frozen at −20°C for subsequent fermentation profile determination. In addition, samples were collected to evaluate DM content, dried and ground as described previously. Undried samples were collected and used to evaluate yeast and mold counts as described previously. After sample collection was completed, remaining silage (approximately 6 kg) for each individual silo was mixed and placed back in the experimental silo with two temperature sensors (HOBO temperature data logger 64 k; Onset Computer Corp., Cape Cod, MA, United States) placed in the geometric center of the bucket to evaluate aerobic stability. Sensors recorded the temperature every 30 min, and three additional sensors were used to monitor room temperature in the event of temperature fluctuations. Room temperature averaged 22.4°C ± 1.14 among all periods of aerobic stability measurements. Buckets were covered with two layers of cheesecloth to prevent drying and silos were left exposed to aerobic conditions for 240 h. Aerobic stability was defined as the number of hours until silo temperature increased 2°C above the baseline silo temperature.

Both, dried, and frozen samples were sent to Rock River Laboratory Inc. (Watertown, WI, United States) for analysis of nutrient composition and fermentation profile. Samples ensiled for 14, 28 and 56 d were analyzed for pH, organic acids, and fermentation byproducts (1,2-PD, 1-propanol, 2,3-butanediol, 2-butanol and ethanol). Twenty grams of undried, unground sample was diluted 10-fold (mass basis) in double distilled water, blended for 30 s in a high-speed blender, and filtered through a filter funnel with a 2-mm pore size screen to extract silage juice. The extract was collected, and pH was immediately measured using a pH meter (Thermo-Orion Dual Star; Thermo Fisher Scientific Inc.) fitted with a glass pH electrode (Thermo-Orion 9172BNWP; Thermo Fisher Scientific Inc.). After pH was measured, the extract was centrifuged (750 × g) for 20 min at 25°C, and the supernatant was combined with 1.0 mL of calcium hydroxide solution and 0.5 mL of copper sulfate solution and re-centrifuged as described previously. The supernatant was analyzed for organic acids, and alcohols using high-performance liquid chromatography with an isocratic pump, auto sampler, column heater, refractive index detector (1515, 2707, heater, and 2414 respectively; Waters Corporation, Milford, MA, United States), and a reverse-phase ion exclusion column (Bio-Rad Aminex HPX-876H; Bio-Rad Laboratories, Hercules, CA). Flow rate and temperature were set at 0.6 mL/min for 40 min and 35°C, respectively. The mobile phase used was 0.015 N H_2_SO_4_/0.25 mM EDTA.

#### Statistical Analysis

Data were analyzed as a completely randomized design with a 2 × 3 factorial arrangement of treatments, with laboratory silos as experimental units, using PROC GLIMMIX of SAS (version 9.4; SAS Institute Inc., Cary, NC, United States) according to the statistical equation:

$$Y_{ij}=\mu +I_{i}+S_{j}+IS_{ij}+e_{ij},$$

with $e_{ij}\approx N\left(0,\sigma_{e}^{2}\right)$, where *Y*_*ij*_ is the value of the dependent variable, μ is the overall mean, *I_i_* is the fixed effect of microbial inoculant (i = 1 and 2), *S_j* is the fixed effect of storage length (j = 1 to 3), *IS*_*ij*_ is the fixed interaction effect, *e*_*ij*_ is the residual error, *N* stand Gaussian distribution, σ^2^_*e*_ is the residual variance. Degrees of freedom were corrected by Kenward and Roger (1997) method. If significant, interaction effects were partitioned using the SLICE option to study the effect of microbial inoculation within each day of storage length. Significance was declared at *P* < 0.05.

### Experiment 2

This experiment was carried out at the University of Florida Plant Science Research and Education Unit (Citra, FL, United States). A completely randomized design with a 2 × 3 factorial arrangement of treatments was used to evaluate the effect of microbial inoculation [CON (50 mL distilled water) or LBLD (Provita Supplements Inc., Mendota Heights, MN; *Lactobacillus plantarum* DSM 21762, *L. buchneri* DSM 12856, and *L. diolivorans* DSM 32074; 300,000 CFU/g of wet forage)] and storage length (14, 28, or 56 d) in WPSS made from forage sorghum, sweet sorghum, and sorghum sudangrass.

#### Crop Establishment, Harvesting, and Ensiling

Forage sorghum, sorghum-sudangrass, and sweet sorghum were planted in July 2019, at 75,000, 122,000, and 75,000 plants.ha^–1^ seeding rates following companies’ specifications, respectively, in an experimental irrigated field of 4 plots (considered as replication). Varieties were seeded in 4 rows of 6 m length each, spaced at 76 cm centers by a John Deere MaxEmerge Plus 170 4-row planter (Moline, IL, United States). Fertilizer applications consisted of 303 kg.ha^–1^ nitrogen, 237 kg.ha^–1^ potassium, 63 kg.ha^–1^ phosphorus, 40 kg.ha^–1^ sulfur, 18 kg.ha^–1^ magnesium, 11 kg.ha^–1^ manganese, 4.5 kg.ha^–1^ zinc divided in starter in furrow, 2 applications side-dressed and a final application applied through overhead irrigation. Pesticide application included 1-Chloro-3-ethylamino-5-isopropylamino-2,4,6-triazine (Atrazine, Syngenta, Lone Tree, IA, United States), Pendimethaline Penoxaline (Prowl, Basf, Research Triangle Park, NC, United States), and Metolachlor (Dual, Syngenta, Lone Tree, IA), at planting for weed control; Tebuconazole (TebuStar, Albaugh LLC, Ankeny, IA, United States) and Pyraclostrobin (Headline, Basf, Research Triangle Park, NC, United States) at 76 cm plant height, and Pyraclostrobin and Metconazole (Headline Amp, Basf, Research Triangle Park, NC, United States) at tasseling for fungal disease control. Insecticide application comprised of Chlorantraniliprole (Coragen, FMC Philadelphia, PA, United States), Cyhalothrin (Besiege, Syngenta, Lone Tree, IA), Lambda-cyhalothrin (Warrior, Syngenta, Lone Tree, IA, United States), and Flubendiamide (Belt, Bayer CropScience, Research Triangle Park, NC, United States) divided into 6 applications, following the guidelines established by the University of Florida/IFAS.

After tasseling, plants were monitored weekly for DM content and harvested at a targeted DM of 32%. Varieties were harvested individually on different days as plants tested at the threshold of 32% DM concentration. All plots from a variety were harvested on the same day. At harvest, in November 2019, plants from the 2 middle rows of each plot were hand-cut to a 25-cm stubble, in a 3-m continuous section and immediately processed with a single-row silage chopper (Model #707 SN: 245797; CNH Industrial America LLC, Burr Ridge, IL, United States) using a theoretical length of cut of 17 mm and without a kernel processor.

Microbial inoculation rates were based on LAB counts determined in experiment 1. Microbial inoculant was mixed and combined with sorghum forage as described before. Approximately 900 g of material was placed in nylon-polyethylene standard barrier vacuum pouches silos (0.09 mm thickness, 25.4 × 35.6 cm; Doug Care Equipment Inc., Springville, CA, United States), which were the experimental units, vacuum-sealed using an external clamp vacuum machine (Bestvac; distributed by Doug Care Equipment), and were randomly assigned to be stored for 14, 28, or 56 d. All silos were filled and sealed within 2 h after harvest, weighed then stored at room temperature in the dark until reaching the assigned storage length. Therefore, the experiment consisted of 6 treatments (two microbial inoculations × three storage lengths) per variety and 72 total mini-silos (four replications per treatment).

#### Chemical Analysis

Fresh samples from each plot of each variety were analyzed for DM, pH and yeast and mold counts as described previously. Additionally, dried samples were ground as described previously, and sent to Rock River Laboratory Inc. (Watertown, WI, United States) for chemical characterization (DM, CP, EE, starch, WSC, NDF, and ash). At opening, silos were weighed, and unground sample of 20 g was diluted 10-fold (mass basis) in 0.1% peptone water (Oxoid CM0090), blended for 60 s in a high-speed stomacher (Lab-Blender 400, Tekmar Company, Cincinnati, OH), and filtered through two layers of cheese cloth to extract forage juice. The extract was collected, and pH was measured as described previously. Forage extract was centrifuged (750 × g) for 20 min at 25°C, and the supernatant (40 mL) was combined with 0.4 mL of 50% sulfuric acid solution and re-centrifuged as previously described. The supernatant was kept in a freezer (−20°C) for subsequent analysis of organic acids and 1,2-PD. The concentrations of organic acids and 1,2-PD were determined using high-performance liquid chromatography (Merck Hitachi Elite La-Chrome, Hitachi L2400, Tokyo, Japan) as described by Muck and Dickerson (1988). For the organic acids analysis, a Bio-Rad Aminex HPX-87H ion exclusion column (300 × 7.8 mm id; Bio-Rad Laboratories, Hercules, CA) was used in an isocratic elution system containing 0.015 M sulfuric acid in the mobile phase of the high-performance liquid chromatography attached to an ultraviolet light detector (wavelength 210 nm; L-2400, Hitachi) using a flow rate of 0.7 mL/min during 40 min at 45°C. For 1,2-PD analysis, the same system and column were used running 0.005 M sulfuric acid in the mobile phase of the high-performance liquid chromatography attached to a Refractive Index detector (L-2490, Hitachi) using a flow rate of 0.6 mL/min during 40 min at 45°C. Unacidified forage juice extract was also evaluated for yeast and mold counts as described in experiment 1. The recovery of DM was calculated through the methodology proposed by Jobim et al. (2007), according to the equation:

$$\mathrm{DM}\mathrm{recovery}=\frac{\left(\text{FO}\times \text{DMO}\right)}{\left(\mathrm{FS}\times \mathrm{DMS}\right)}$$

where FO is the forage weight at opening, DMO is the dry matter concentration of the forage at opening, FS is the initial forage weight during ensiling, DMS is the initial dry matter concentration of the forage during ensiling.

A silage sample of 50 g was dried in a forced-air oven set at 60°C for 48 h and ground as described previously. Fermented samples were sent to Rock River Laboratory Inc. (Watertown, WI) for chemical analysis. Samples were analyzed for absolute DM, CP, EE, starch, WSC, ash and NDF as described previously, as well as borate-phosphate-soluble CP (SP; Krishnamoorthy et al., 1982).

Additional dried silage samples were ground to pass through a 6-mm sieve using a Wiley Mill for ruminal in situ NDF disappearance. Ruminal *in situ* procedures were conducted at the University of Florida (Gainesville, FL, United States) under a protocol approved by the University of Florida, Institute of Food and Agricultural Sciences, and Animal Care Research Committee (protocol #201709849). A sample of 5 g was placed at Dacron polyester cloth bags (R1020, 10 × 20 cm and 50 ± 10 microporosity; Ankom Technology, Macedon, NY, United States) and incubated for 30 h in the rumen of three ruminally cannulated, mid-lactation, multiparous Holstein cows. Cows were fed a diet containing (DM basis) corn silage (38.2%), alfalfa hay (4.0%), dry ground shelled corn (27.3%), soybean meal (14.5%), citrus pulp (9.1%), minerals and supplements (6.8%). Additionally, two empty bags were incubated for correction of bag infiltration or losses. After the incubation period, bags were removed from the rumen, soaked in cold water for approximately 15 min to stop microbial activity, rinsed in a washing machine using the rinse mode and spin cycle, set with room temperature water for a 30-min cycle (Roper RTW4516F^∗^, Whirlpool Corp., Benton Harbor, MI, United States), dried in a forced-air oven at 60° C for 72 h, and initial and residual NDF contents were analyzed with the percentage of difference considered as NDF disappearance.

#### Statistical Analysis

Data were analyzed separately for each variety as a completely randomized design with a 2 × 3 factorial arrangement of treatments with laboratory silos as the experimental units and using the PROC GLIMMIX procedure of SAS (version 9.4; SAS Institute Inc, Cary, NC, United States) according to the statistical equation:

$$Y_{ijk}=\mu +I_{j}+S_{k}+IS_{jk}+e_{ijk},$$

with $e_{ijk}\approx N\left(0,\sigma_{e}^{2}\right)$, where *Y*_*ijk*_ is the value of the dependent variable, μ is the overall mean,, *I_j_* is the fixed effect of microbial inoculant (*j* = 1 and 2), *S_k* is the fixed effect of storage length (*k* = 1 to 3), *IS*_*jk*_, is the fixed interaction effect, *e*_*ijk*_ is the residual error, *N* stand Gaussian distribution, σ${}_{e}^{2}$ is the residual variance. Degrees of freedom were corrected by Kenward and Roger (1997) method. If significant, interaction effects were partitioned using the SLICE option to study the effect of microbial inoculation within each day of storage length. Significance was declared at *P* < 0.05.

## Results

Main effects are discussed only if there were no significant interaction effects detected.

### Experiment 1

Nutrient composition, pH, and yeast and mold counts of fresh sorghum forage are presented in Table 1. Effects of microbial inoculation and storage length on fermentation profile of whole-plant sorghum silage are in Table 2. Concentrations of butyric acid and 2-butanol were not detected among any treatments. The pH of silage was affected by microbial inoculant and was lower (*P* = 0.001) in LBLD treated silages (3.86) than CON (3.95). Total acid concentration was affected by microbial inoculant and storage length (*P* ≤ 0.01). Total acid concentration was 11.5% DM for LBLD silage, while CON was 10.6% DM. Additionally, total acid concentration was greater after 56 d (12.5 % DM) compared to other storage lengths (10.3% DM, on average). An interaction between microbial inoculant × storage length was observed (*P* = 0.05) for the concentration of lactic acid, which was similar between microbial inoculants at 14 and 28 d, but lower for LBLD than CON after 56 d of storage. Similarly, acetic acid concentration was affected by the interaction of microbial inoculant × storage length (*P* = 0.01), with similar concentrations in LBLD and CON silages after 14 d of storage (1.5% of DM, on average), but greater for LBLD compared to CON silage after 28 (2.1 vs. 1.3% of DM, respectively) and 56 d (4.0 vs. 1.7% of DM, respectively). Succinic acid was affected by microbial inoculant and was greater (*P* = 0.02) for LBLD (0.12% DM) than CON silage (0.08% DM). No fixed or interaction effects (*P* ≥ 0.16) were observed for propionic acid concentration.

**TABLE 1:** Nutrient composition, pH, and yeast and mold counts of fresh, uninoculated whole-plant sorghum forage in experiments 1 and 2.

**Item^1^**	**Experiment1**	**Experiment 2**		
	**Forage sorghum**	**Sorghum-sudangrass**	**Sweet sorghum**	**Forage sorghum**
DM, % of as fed	22.1 ± 0.60	34.9 ± 1.70	30.8 ± 1.75	30.6 ± 1.49
pH	5.84 ± 0.10	5.65 ± 0.19	5.95 ± 0.25	6.30 ± 0.08
WSC, % DM	11.2 ± 2.08	12.1 ± 1.13	34.1 ± 0.87	14.9 ± 2.17
CP, % DM	6.6 ± 0.33	6.5 ± 0.59	5.0 ± 1.84	7.9 ± 0.23
EE, % DM	2.9 ± 0.19	2.6 ± 0.80	1.6 ± 0.70	2.6 ± 0.38
NDF, % DM	59.4 ± 1.49	64.7 ± 1.21	49.2 ± 0.32	49.7 ± 1.17
Starch, % DM	4.10 ± 0.87	5.10 ± 0.79	15.3 ± 4.27	21.2 ± 0.79
Ash, % DM	5.90 ± 0.96	4.04 ± 0.20	2.97 ± 0.40	3.49 ± 0.21
Yeast, log CFU/g	4.18 ± 0.79	8.62 ± 0.21	9.36 ± 0.35	10.90 ± 0.42
Mold, log CFU/g	4.30 ± 0.51	2.20 ± 4.40	6.00 ± 4.00	2.90 ± 3.80

*^1^DM, dry matter; WSC, water-soluble carbohydrates; CP, crude protein; EE, ether extract; NDF, neutral detergent fiber.*

**TABLE 2:** Effect of microbial inoculation and storage length on the fermentation profile of whole-plant sorghum silage in experiment 1.

**Item^1^**	**pH**	**Total acids, % DM**	**Lactic acid, % DM**	**Acetic acid, % DM**	**Propionic acid, % DM**	**Succinic acid, % DM**	**Ethanol, % DM**	**1,2 propanediol, % DM**	**1 propanol, % DM**	**2,3 butanediol, % DM**
d 14										
CON	3.96	10.2	8.8	1.3	0.1	0.09	0.7	0.0	0.0	0.0
LBLD	3.88	10.6	8.5	1.8	0.2	0.11	1.3	0.1	0.0	0.1
d 28										
CON	3.94	9.6	8.2	1.3^b^	0.1	0.08	0.9	0.0	0.0	0.0
LBLD	3.86	10.8	8.4	2.1^a^	0.2	0.12	1.4	0.7	0.2	0.2
d 56										
CON	3.94	11.9	10.0^a^	1.7^b^	0.1	0.09	0.6	0.0	0.0^b^	0.0
LBLD	3.84	13.0	8.5^b^	4.0^a^	0.4	0.14	1.4	0.8	0.6^a^	0.4
Storage length means										
d 14	3.92	10.4^z^	8.6	1.5	0.1	0.11	1.0	0.1	0.0	0.1
d 28	3.90	10.2^z^	8.3	1.7	0.1	0.10	1.1	0.3	0.1	0.1
d 56	3.89	12.5^y^	9.2	2.9	0.3	0.11	1.0	0.4	0.3	0.2
Microbial inoculation means										
CON	3.95	10.6	9.0	1.4	0.1	0.08	0.7	0.0	0.0	0.0
LBLD	3.86	11.5	8.4	2.7	0.2	0.12	1.4	0.5	0.3	0.2
SEM^2^	0.02	0.41	0.35	0.35	0.17	0.03	0.31	0.33	0.11	0.09
*P*-value										
SL	0.26	0.001	0.02	0.001	0.51	0.71	0.75	0.31	0.01	0.06
MI	0.001	0.01	0.06	0.001	0.16	0.02	0.01	0.01	0.001	0.001
SL × MI	0.83	0.47	0.05	0.001	0.73	0.75	0.63	0.31	0.01	0.06

*^*a,b*^Means with different superscripts within the same day denote an effect of microbial inoculation within that day.*

*^y,z^Means with different superscripts denote differences among storage lengths.*

*^1^MI, effect of microbial inoculant (50 ml of distilled water or 300,000 CFU/g wet forage of *L. plantarum* DSM 21762, *L. buchneri* DSM 12856, and *L. diolivorans* DSM 32074 [Provita Supplements Inc., Mendota Heights, MN]); SL, effect of storage length (14, 28, and 56 d).*

*^2^Greatest standard error of the mean.*

Both the concentration of ethanol (*P* = 0.01; 1.4% and 0.7% DM, respectively) and 1,2-PD (*P* = 0.01; 0.5% and 0.0% DM, respectively) were greater for LBLD silages compared to CON silages. Additionally, an interaction between microbial inoculant × storage length was observed for 1-propanol. There was no 1-propanol detected at 14 d for any treatment, while LBLD treated silage had (*P* = 0.01) 0.6% DM after 56 d of storage. The concentration of 2,3-butanediol was affected by microbial inoculant and was greater (*P* = 0.001) for LBLD (0.2 % DM) than CON silages (0.0% DM).

Effects of microbial inoculation and storage length on nutrient composition, yeast count, and aerobic stability of whole-plant sorghum silage are in Table 3. Starch concentration and mold counts were also evaluated. For starch, because a male sterilized sorghum variety was used for this experiment, all treatment averages were less than 1% DM (0.68% DM, on average) and no fixed or interaction effects were observed (*P* > 0.05). Additionally, mold counts were lower than 2 log CFU/g. Therefore, these variables are not included in tables or figures. An interaction between microbial inoculant and storage length was observed (*P* = 0.01) for aerobic stability. Aerobic stability was similar between microbial inoculant treatments at 14 d, but 47 h and 156 h longer for LBLD treated silage than CON after 28 and 56 d of storage, respectively.

**TABLE 3:** Effect of microbial inoculation and storage length on the nutrient composition, yeast counts, and aerobic stability of whole-plant sorghum silage in experiment 1.

**Item^1^**	**DM, % as fed**	**Ash, % DM**	**WSC, % DM**	**EE, % DM**	**CP, % DM**	**NDF, % DM**	**Yeast, log CFU/g**	**Aerobic stability, h**
d 14								
CON	21.3	6.9	2.5	2.9	7.3	48.5	2.65	136
LBLD	21.2	5.6	3.0	3.0	6.9	61.8	5.40	157
d 28								
CON	21.1^a^	7.6	1.9	2.7	7.3	62.2	3.88	76^b^
LBLD	20.4^b^	6.2	2.6	2.9	7.6	61.5	2.47	123^a^
d 56								
CON	21.2^a^	6.9	1.9	3.2	7.1	61.1	5.08	35^b^
LBLD	19.6^b^	6.6	2.5	3.1	7.5	64.4	3.14	191^a^
Storage length means								
d 14	21.3	6.2	2.7	3.0	7.1	55.1	4.03	147
d 28	20.8	6.9	2.2	2.8	7.4	61.8	3.18	100
d 56	20.4	6.8	2.2	3.1	7.3	62.7	4.11	113
Microbial inoculation means								
CON	21.2	7.1	2.1	2.9	7.2	57.3	3.88	82
LBLD	20.4	6.1	2.7	3.0	7.3	62.5	3.67	156
SEM^1^	0.32	0.46	0.57	0.21	0.23	5.82	1.46	24.59
*P*-Value								
SL	0.01	0.12	0.49	0.11	0.26	0.37	0.62	0.04
MI	0.001	0.01	0.16	0.43	0.46	0.28	0.82	0.001
SL × MI	0.01	0.25	0.99	0.61	0.07	0.47	0.07	0.01

*^*a,b*^ Means with different superscripts within the same day denote an effect of microbial inoculation within that day.*

*^1^MI: effect of microbial inoculant (50 ml of distilled water or 300,000 CFU/g wet forage of *L. plantarum* DSM 21762, *L. buchneri* DSM 12856, and *L. diolivorans* DSM 32074 [Provita Supplements Inc., Mendota Heights, MN]); SL: effect of storage length (14, 28, and 56 d).*

*^2^ Greatest standard error of the mean.*

Overall, microbial inoculant and storage length had little effect on the nutrient composition of whole-plant sorghum silage, with no effects (*P* > 0.07) observed for WSC, EE, CP, and NDF concentrations in addition to yeast counts. An interaction between microbial inoculant and storage length was observed (*P* = 0.01) for DM. Concentration of DM was similar at 14 d, but slightly greater (1.0%-unit, on average) for CON than LBLD at 28 and 56 d of storage. A microbial inoculant effect (*P* = 0.01) was observed for ash concentration which was greater for CON (7.1% DM) than LBLD silage (6.1% DM).

### Experiment 2

Nutrient composition, pH, and yeast and mold counts of fresh, uninoculated sorghum varieties are in Table 1. Furthermore, similar to experiment 1, because all mold counts were less than 2 log CFU/g, this variable is not included in any tables.

Effects of microbial inoculation and storage length on fermentation profile of whole-plant forage sorghum silage are in Table 4. No fixed or interaction effects (*P* ≥ 0.20) were observed for silage pH. Lactic, acetic, and butyric acids in addition to 1,2-PD were affected by an interaction of microbial inoculant × storage length (*P* ≤ 0.05). Lactic acid concentration was greater (11.3% DM) for CON silage than LBLD (4.7% DM) at 14 d of storage, but similar (10% DM, on average) at 28 and 56 d. Similarly, acetic acid concentration for CON silage was greater (2.6% vs. 1.0% DM, respectively) at 14 d, similar at 28 d, but lower (1.7% vs. 5.7% DM, respectively) than LBLD after 56 d of storage. Likewise, butyric acid only had detectable concentrations for CON silages after 14 d (0.5% DM) and 28 d (0.3% DM) of storage. The concentration of 1,2-PD was greater for LBLD silage than CON after 28 (0.9 and 0.0% DM, respectively) and 56 d (3.1 and 0.0% DM, respectively) of storage, but not 14 d. For propionic acid, no treatments had detectable concentration except for CON silage after 56 d (0.7% DM).

**TABLE 4:** Effect of microbial inoculation and storage length on the fermentation profile of whole-plant forage sorghum silage in experiment 2.

**Item^1^**	**pH**	**Lactic acid, % DM**	**Acetic acid, % DM**	**Propionic acid, % DM**	**Butyric acid, % DM**	**1,2 propanediol, % DM**
d 14						
CON	3.54	11.3^a^	2.6^a^	0.0	0.5^a^	0.0
LBLD	3.51	4.7^b^	1.0^b^	0.0	0.0^b^	0.3
d 28						
CON	3.50	11.2	2.5	0.0	0.3^a^	0.0^b^
LBLD	3.50	12.3	3.5	0.0	0.0^b^	0.9^a^
d 56						
CON	3.60	9.6	1.7^b^	0.7	0.0	0.0^b^
LBLD	3.65	8.7	5.7^a^	0.0	0.0	3.1^a^
Storage length means						
d 14	3.53	8.0	1.8	0.0	0.2	0.2
d 28	3.50	11.8	3.0	0.0	0.2	0.4
d 56	3.62	9.1	3.7	0.3	0.0	1.6
Microbial inoculation means						
CON	3.55	10.7	2.3	0.2	0.3	0.0
LBLD	3.55	8.6	3.4	0.0	0.0	1.4
SEM^2^	0.07	0.86	0.38	−	0.10	0.23
*P*-value						
SL	0.20	0.001	0.001	−	0.08	0.0001
MI	0.92	0.01	0.01	−	0.08	0.0001
SL × MI	0.85	0.001	0.001	−	0.05	0.0001

*^*a,b*^ Means with different superscripts within the same day denote an effect of microbial inoculation within that day.*

*^1^MI, effect of microbial inoculant (50 ml of distilled water or 300,000 CFU/g wet forage of *L. plantarum* DSM 21762, *L. buchneri* DSM 12856, and *L. diolivorans* DSM 32074 [Provita Supplements Inc., Mendota Heights, MN]); SL: effect of storage length (14, 28, and 56 d).*

*^2^Greatest standard error of the mean.*

Effects of microbial inoculation and storage length on the nutrient composition of whole-plant forage sorghum are presented in Table 5. Concentration of DM was greater (*P* = 0.04) at 14 d of storage length (29.2% as fed) than 28 and 56 d (27.6% as fed, on average). Similarly, ash concentration was greater (*P* = 0.01) at 28 d (4.1% DM) than other storage length treatments (3.6% DM, on average). An interaction between microbial inoculant × storage length (*P* = 0.05) was observed for WSC concentration; CON silage (3.6% DM) was greater than LBLD (1.7% DM) at 28 d, but similar at 14 and 56 d. The concentration of EE was greater (*P* = 0.001) for LBLD (2.4% DM) than CON silage (1.6% DM). Soluble CP concentration was affected by both microbial inoculation (*P* = 0.001) and storage length (*P* = 0.02). Soluble CP was greater in CON silage (53.2% DM) compared with LBLD (46.1% DM). Additionally, the concentration of soluble CP was lower after 14 d of storage (46.3% DM) compared with other storage lengths (51.3% DM, on average). Starch concentration was affected by storage length and was lower (*P* = 0.01) after 28 d of storage (19.2% DM) compared with other storage lengths (23.7% DM, on average). No fixed or interaction effects (*P* ≥ 0.08) were observed for DM recovery, concentrations of CP and NDF, NDF disappearance and yeast counts.

**TABLE 5:** Effect of microbial inoculation and storage length on the nutrient composition and yeast counts of whole-plant forage sorghum silage in experiment 2.

**Item^1^**	**DM, % as fed**	**DM recovery, % DM**	**Ash, % DM**	**WSC, % DM**	**EE, % DM**	**CP, % DM**	**Sol.CP, % CP**	**NDF, % DM**	**Starch, % DM**	**NDF disappearance, % NDF**	**Yeast, log CFU/g**
d 14											
CON	29.7	97.4	3.3	3.8	1.4	8.7	48.6	46.8	22.8	26.4	0.60
LBLD	28.7	94.8	3.7	3.7	2.1	8.3	44.0	46.3	24.0	25.4	0.85
d 28											
CON	27.6	90.6	4.1	3.6^a^	1.5	8.4	55.4	49.3	18.5	22.0	0.64
LBLD	27.5	91.5	4.1	1.7^b^	2.7	8.5	46.4	50.8	19.9	28.2	1.03
d 56											
CON	27.3	90.1	3.9	2.1	2.0	8.4	55.5	44.9	22.4	22.1	0.00
LBLD	27.9	92.2	3.5	2.7	2.5	8.9	47.8	48.3	25.8	24.3	0.00
Storage length means											
d 14	29.2^y^	96.1	3.5^z^	3.8	1.7	8.5	46.3^z^	46.6	23.4^y^	25.9	0.72
d 28	27.5^z^	91.0	4.1^y^	2.7	2.1	8.5	50.9^y^	50.0	19.2^z^	25.1	0.84
d 56	27.6^z^	91.2	3.7^z^	2.4	2.2	8.7	51.7^y^	46.6	24.1^y^	23.2	0.00
Microbial inoculation means											
CON	28.2	92.7	3.8	3.2	1.6	8.5	53.2	47.0	21.2	23.5	0.41
LBLD	28.0	92.8	3.8	2.7	2.4	8.6	46.1	48.5	23.3	26.0	0.62
SEM^1^	0.68	2.58	0.14	0.49	0.22	0.22	1.79	1.66	1.42	2.26	0.60
*P*-value											
SL	0.04	0.11	0.01	0.03	0.12	0.56	0.02	0.08	0.01	0.48	0.34
MI	0.79	0.95	0.93	0.26	0.001	0.63	0.001	0.30	0.10	0.19	0.67
SL × MI	0.51	0.63	0.07	0.05	0.37	0.15	0.47	0.52	0.71	0.30	0.95

*^*a,b*^ Means with different superscripts within the same day denote an effect of microbial inoculation within that day.*

*^*y,z*^ Means with different superscripts denote differences among storage lengths.*

*^1^MI: effect of microbial inoculant (50 ml of distilled water or 300,000 CFU/g wet forage of *L. plantarum* DSM 21762, *L. buchneri* DSM 12856, and *L. diolivorans* DSM 32074 [Provita Supplements Inc., Mendota Heights, MN]); SL: effect of storage length (14, 28, and 56 d).*

*^2^ Greatest standard error of the mean.*

Effects of microbial inoculation and storage length on fermentation profile of whole-plant sweet sorghum silage are in Table 6. An interaction between microbial inoculant × storage length (*P* = 0.001) was observed for pH, which was greater for CON (3.69) than LBLD (3.24) at 14 d of storage, but similar between microbial inoculants at 28 and 56 d. Lactic acid concentration was affected by microbial inoculant and storage length (*P* ≤ 0.04). Lactic acid concentration was 7.9% DM for LBLD silage, while CON was 10.1% DM. Moreover, lactic acid concentration was lower after 14 d (5.6% DM) in comparison to other storage lengths (10.7% DM, on average). An interaction between microbial inoculant × storage length (*P* ≤ 0.03) was observed for acetic, propionic, and butyric acids along with 1,2-PD. Concentration of acetic acid did not differ between microbial inoculants at 14 and 28 d but was greater for LBLD (5.0% DM) compared with CON (3.7% DM) at 56 d. Propionic acid concentration did not differ at 14 d, but was greater for CON than LBLD treatment at 28 (0.5 vs. 0.0% of DM, respectively) and 56 d (0.6 vs. 0.0% of DM, respectively). The butyric acid concentration for CON (0.7% DM) was greater than LBLD (0.0% DM) silage at 14 d, but not at 28 and 56 d. No differences were detected for 1,2-PD concentration at 14 d, while LBLD was greater than CON silage after 28 (0.5 vs. 0.0 % of DM, respectively) and 56 d (3.1 vs. 0.0% of DM, respectively) of storage.

**TABLE 6:** Effect of microbial inoculation and storage length on the fermentation profile of whole-plant sweet sorghum silage in experiment 2.

**Item^1^**	**pH**	**Lactic Acid, % DM**	**Acetic Acid, % DM**	**Propionic Acid, % DM**	**Butyric Acid, % DM**	**1,2 Propanediol, % DM**
d 14						
CON	3.69^a^	7.2	2.3	0.0	0.7^a^	0.0
LBLD	3.24^b^	4.1	1.2	0.2	0.0^b^	0.0
d 28						
CON	3.35	11.2	3.9	0.5^a^	0.0	0.0^b^
LBLD	3.29	9.5	3.7	0.0^b^	0.0	0.5^a^
d 56						
CON	3.30	11.9	3.7^b^	0.6^a^	0.0	0.0^b^
LBLD	3.40	10.3	5.0^a^	0.0^b^	0.0	3.1^a^
Storage length means						
d 14	3.46	5.6^z^	1.8	0.1	0.3	0.0
d 28	3.32	10.3^y^	3.8	0.2	0.0	0.3
d 56	3.35	11.1^y^	4.4	0.3	0.0	1.5
Microbial inoculation means						
CON	3.45	10.1	3.3	0.4	0.2	0.0
LBLD	3.31	7.9	3.3	0.1	0.0	1.2
SEM^2^	0.04	1.20	0.41	0.10	0.01	0.15
*P*-value						
SL	0.01	0.001	0.001	0.06	0.001	0.001
MI	0.001	0.04	0.98	0.001	0.001	0.001
SL × MI	0.001	0.77	0.03	0.001	0.001	0.001

*^*a,b*^ Means with different superscripts within the same day denote an effect of microbial inoculation within that day.*

*^*y,z*^ Means with different superscripts denote differences among storage lengths.*

*^1^MI: effect of microbial inoculant (50 ml of distilled water or 300,000 CFU/g wet forage of *L. plantarum* DSM 21762, *L. buchneri* DSM 12856, and *L. diolivorans* DSM 32074 [Provita Supplements Inc., Mendota Heights, MN]); SL: effect of storage length (14, 28, and 56 d).*

*^2^Greatest standard error of the mean.*

Effects of microbial inoculation and storage length on nutrient composition and yeast count of whole-plant sweet sorghum silage are in Table 7. Overall, microbial inoculant and storage length had minimal effects on the nutrient composition of whole-plant sweet sorghum silage, with no fixed or interaction effects (*P* ≥ 0.09) observed for DM, DM recovery, concentrations of ash and soluble CP, and NDF disappearance. An interaction between microbial inoculant × storage length (*P* ≤ 0.02) was observed for WSC and EE concentrations. Water-soluble carbohydrates concentrations were similar between microbial inoculants at 14 and 28 d, but greater for CON (21.4% DM) than LBLD silage (15.6% DM) at 56 d. The EE concentration of LBLD was greater than CON at 14 (1.7 vs. 0.9% DM, respectively) and 28 d (1.8 vs. 1.0% DM, respectively), but did not differ at 56 d. A microbial inoculant effect (*P* = 0.02) was observed for CP concentration which was greater for LBLD (4.4% DM) than CON silage (4.0% DM). A storage length effect (*P* = 0.03) was observed for NDF concentration, which was lower after 14 d (45.9% DM) compared with other storage lengths (49.1% DM, on average). Similarly, starch concentration was affected by storage length (*P* = 0.02) and was greater at 14 d (11.7% DM) than 28 and 56 d (9.3% DM, on average). Furthermore, yeast counts were affected by microbial inoculation (*P* = 0.001) and were greater for CON (2.8 log CFU/g) than LBLD silage (0.0 log CFU/g).

**TABLE 7:** Effect of microbial inoculation and storage length on the nutrient composition and yeast counts of whole-plant sweet sorghum silage in experiment 2.

**Item^1^**	**DM, % as fed**	**DM recovery, % DM**	**Ash, % DM**	**WSC, % DM**	**EE, % DM**	**CP, % DM**	**Sol.CP, % CP**	**NDF, % DM**	**Starch, % DM**	**NDF disappearance, % NDF**	**Yeast, log CFU/g**
d 14											
CON	26.9	93.5	3.0	22.5	0.9^b^	4.0	65.5	46.5	11.7	22.2	2.1
LBLD	26.9	93.4	3.2	22.3	1.7^a^	4.1	58.2	45.3	11.6	23.1	0.0
d 28											
CON	27.0	92.2	3.2	19.2	1.0^b^	4.0	59.6	48.3	9.5	23.0	3.7
LBLD	26.9	93.3	3.2	18.0	1.8^a^	4.3	62.1	50.1	9.1	23.9	0.0
d 56											
CON	27.2	93.8	3.2	21.4^a^	2.1	4.0	66.5	46.3	10.8	22.9	2.4
LBLD	26.0	89.7	3.1	15.6^b^	1.7	4.9	63.9	51.5	7.7	21.1	0.0
Storage length means											
d 14	26.9	93.5	3.1	22.4	1.3	4.0	61.9	45.9^z^	11.7^y^	22.7	1.1
d 28	26.9	92.8	3.3	18.6	1.4	4.2	60.9	49.2^y^	9.3^z^	23.4	1.9
d 56	26.6	91.8	3.2	18.5	1.9	4.5	65.2	48.9^y^	9.2^z^	22.0	1.2
Microbial inoculation means											
CON	27.1	93.2	3.1	21.0	1.3	4.0	64.0	47.0	10.6	22.7	2.8
LBLD	26.6	92.2	3.3	18.6	1.7	4.4	61.4	49.0	9.5	22.7	0.0
SEM^1^	0.65	1.16	0.22	0.97	0.19	0.21	2.15	1.27	0.86	2.37	0.85
*P-Value*											
SL	0.88	0.36	0.62	0.001	0.01	0.13	0.14	0.03	0.02	0.83	0.61
MI	0.42	0.29	0.46	0.01	0.01	0.02	0.17	0.08	0.11	0.99	0.001
SL × MI	0.59	0.09	0.85	0.02	0.01	0.16	0.10	0.07	0.19	0.81	0.61

*^*a,b*^ Means with different superscripts within the same day denote an effect of microbial inoculation within that day.*

*^*y,z*^ Means with different superscripts denote differences among storage lengths.*

*^1^MI: effect of microbial inoculant (50 ml of distilled water or 300,000 CFU/g wet forage of *L. plantarum* DSM 21762, *L. buchneri* DSM 12856, and *L. diolivorans* DSM 32074 [Provita Supplements Inc., Mendota Heights, MN]); SL: effect of storage length (14, 28, and 56 d).*

*^2^Greatest standard error of the mean.*

Effects of microbial inoculation and storage length on the fermentation profile of whole-plant sorghum sudangrass silage are presented in Table 8. Propionic acid concentration was evaluated, but not detected among any treatments. No fixed or interaction effects (*P ≥* 0.09) were observed for silage pH. An interaction of microbial inoculant × storage length was detected (*P* ≤ 0.05) for lactic, acetic, and butyric acids as well as 1,2-PD concentration. Lactic acid concentration was greater for CON (9.3% DM) than LBLD (4.8% DM) silage at 14 d, but similar at 28 and 56 d. Acetic acid concentration was greater for CON silage in comparison to LBLD at 14 d (2.5 vs. 1.1% of DM, respectively), similar at 28 d, but lower at 56 d (2.0 vs. 3.3% of DM, respectively). The concentration of butyric acid was greater for CON (0.6% DM) than LBLD (0.0% DM) silage at 14 d but did not differ at 28 and 56 d. The 1,2-PD concentration was greater for LBLD silage than CON after 28 (0.3 vs. 0.0 % of DM, respectively) and 56 d (0.6 vs. 0.0 % of DM, respectively).

**TABLE 8:** Effect of microbial inoculation and storage length on the fermentation profile of whole-plant sorghum-sudangrass silage in experiment 2.

**Item^1^**	**pH**	**Lactic acid, % DM**	**Acetic acid, % DM**	**Butyric acid, % DM**	**1,2 propanediol, % DM**
d 14					
CON	3.83	9.3^a^	2.5^a^	0.6^a^	0.0
LBLD	3.69	4.8^b^	1.1^b^	0.0^b^	0.2
d 28					
CON	3.74	7.3	1.7	0.3	0.0^b^
LBLD	3.73	6.8	1.5	0.1	0.3^a^
d 56					
CON	3.78	7.6	2.0^b^	0.0	0.0^b^
LBLD	3.64	9.4	3.3^a^	0.0	0.6^a^
Storage length means					
d 14	3.76	7.1	1.8	0.3	0.1
d 28	3.74	7.0	1.6	0.2	0.2
d 56	3.71	8.5	2.6	0.0	0.3
Microbial inoculation means					
CON	3.79	8.1	2.0	0.3	0.0
LBLD	3.69	7.0	2.0	0.04	0.4
SEM^2^	0.07	1.19	0.45	0.09	0.08
*P-Value*					
SL	0.80	0.39	0.08	0.01	0.03
MI	0.09	0.28	0.93	0.01	0.001
SL × MI	0.60	0.05	0.02	0.01	0.03

*^*a,b*^Means with different superscripts within the same day denote an effect of microbial inoculation within that day.*

*^1^MI: effect of microbial inoculant (50 ml of distilled water or 300,000 CFU/g wet forage of *L. plantarum* DSM 21762, *L. buchneri* DSM 12856, and *L. diolivorans* DSM 32074 [Provita Supplements Inc., Mendota Heights, MN]); SL: effect of storage length (14, 28, and 56 d).*

*^2^Greatest standard error of the mean.*

Effects of microbial inoculation and storage length on the nutrient composition of whole-plant sorghum sudangrass silage are presented in Table 9. The concentration of EE was lower (*P* = 0.01) in CON (2.4% DM) compared with LBLD (2.8% DM) silage. An interaction between microbial inoculant × storage length (*P* = 0.03) was only observed for NDF concentration, which was similar between treatments at 14 and 28 d, but greater in LBLD silage after 56 d (65.4% DM) compared with CON (59.7% DM). Yeast counts were affected by both microbial inoculant (*P* = 0.05) and storage length (*P* = 0.02). Yeast counts were greater in CON silage (2.0 CFU/g) than LBLD silage (0.7 CFU/g). Additionally, yeast counts decreased as storage length increased, from 2.2 CFU/g after 14 d to 0.0 CFU/g after 56 d of storage. No fixed or interaction effects (*P* ≥ 0.06) were observed for DM recovery, concentrations of DM, ash, WSC, CP, soluble CP, and starch, or NDF disappearance.

**TABLE 9:** Effect of microbial inoculation and storage length on the nutrient composition and yeast counts of whole-plant sorghum-sudangrass silage in experiment 2.

**Item^1^**	**DM, % as fed**	**DM Recovery, % DM**	**Ash, % DM**	**WSC, % DM**	**EE, % DM**	**CP, % DM**	**Sol.CP, % CP**	**NDF, % DM**	**Starch, % DM**	**NDF disappearance, % NDF**	**Yeast, log CFU/g**
d 14											
CON	34.6	98.4	4.7	2.8	2.3	7.1	49.3	62.4	4.7	20.2	3.0
LBLD	33.7	97.4	4.6	2.2	2.6	7.0	50.4	61.1	4.2	21.4	1.4
d 28											
CON	33.8	96.9	4.4	3.6	2.3	6.8	57.5	61.8	3.8	24.9	2.9
LBLD	34.2	98.2	4.7	2.0	2.6	7.0	51.6	60.5	4.5	18.5	0.6
d 56											
CON	35.0	99.2	4.4	3.1	2.4	7.2	53.4	59.7^b^	5.3	19.8	0.0
LBLD	34.2	96.6	5.0	2.5	3.2	7.2	54.0	65.4^a^	4.8	18.3	0.0
Storage length means											
d 14	34.2	97.9	4.6	2.5	2.5	7.0	49.9	61.8	4.5	20.8	2.2^y^
d 28	34.0	97.5	4.6	2.8	2.5	6.9	54.6	61.2	4.1	21.7	1.8^y^
d 56	34.6	97.9	4.7	2.8	2.8	7.2	53.7	62.5	5.1	19.0	0.0^z^
Microbial inoculation means											
CON	34.5	98.1	4.5	3.2	2.4	7.0	53.4	61.3	4.6	21.6	2.0
LBLD	34.0	97.4	4.8	2.2	2.8	7.1	52.0	62.3	4.5	19.4	0.7
SEM^2^	0.71	0.85	0.16	0.58	0.18	0.35	2.08	1.40	0.71	1.95	0.76
*P-Value*											
SL	0.72	0.89	0.64	0.82	0.14	0.68	0.08	0.63	0.44	0.40	0.02
MI	0.47	0.29	0.08	0.06	0.01	0.87	0.41	0.39	0.85	0.18	0.05
SL × MI	0.58	0.10	0.14	0.63	0.40	0.83	0.20	0.03	0.62	0.17	0.33

*^*a,b*^Means with different superscripts within the same day denote an effect of microbial inoculation within that day.*

*^*y,z*^Means with different superscripts denote differences among storage lengths.*

*^1^MI: effect of microbial inoculant (50 ml of distilled water or 300,000 CFU/g wet forage of *L. plantarum* DSM 21762, *L. buchneri* DSM 12856, and *L. diolivorans* DSM 32074 [Provita Supplements Inc., Mendota Heights, MN]); SL: effect of storage length (14, 28, and 56 d).*

*^2^Greatest standard error of the mean.*

## Discussion

Both LBLD and CON silages in each experiment had sufficient concentrations of lactic acid for proper forage preservation (Kung et al., 2018). These same authors reported that lower DM silages have the potential to produce greater concentrations of lactic acid, explaining the high concentrations observed in experiment 1. Interestingly, the concentration of lactic acid production observed in CON silages suggests that the epiphytic microbial population had an adequate amount of lactic acid bacteria to promote a beneficial fermentation in all CON treatments for both experiments.

In the current experiments, greater acetic acid concentration in experiment 1 combined with the main effect of inoculation in experiment 1 and interaction effects in experiment 2 on 1,2-PD for LBLD silage compared to CON suggests that *L. buchneri* DSM 12856 was actively involved in a heterofermentative activity after a short period of storage. However, overall, it appears there was a less pronounced heterofermentative activity of *L. diolivorans* DSM 32074, as evidenced by the lack of effect of inoculation on propionic acid in experiment 1. Conversely, because of the heterofermentative activity of the inoculant, inoculating with *L. plantarum* DSM 21762, *L. buchneri* DSM 12856, and *L. diolivorans* DSM 32074 increased total acid production in sorghum silage in experiment 1. Thus, LBLD silages showed a lower pH compared to CON. However, in experiment 2, pH was only affected by an interaction of microbial inoculant and storage length in sweet sorghum. Nonetheless, sweet sorghum pH only differed with microbial inoculant after 14 d of storage, further supporting that in general, CON treatments had sufficient epiphytic LAB to promote a desirable fermentation. Kleinschmit and Kung (2006) reported that the addition of *L. buchneri* might lead to a 0.1 to 0.2 pH units increase due to a conversion of lactic acid to acetic acid, 1,2-PD, and ethanol (Oude Elferink et al., 2001). Previous research (EFSA FEEDAP Panel, 2016) reported that inoculation with *L. diolivorans* alone reduced pH in silages from several different forages, corroborating our results from experiment 1. Increasing the production of total acids, such as in experiment 1, may decrease pH despite LBLD not having a greater lactic acid content, which is the strongest acid in the silo. Moreover, in experiment 1 storage length also increased total acid concentration, demonstrating that the extent of fermentation occurring in the silo increases with time.

In both experiments, LBLD silages had greater concentrations of 1,2-PD compared to CON silages. This is primarily due to the heterofermentative activity of *L. buchneri*, which can produce 1,2-PD by degrading lactic acid while also creating ethanol as a byproduct of this process (Oude Elferink et al., 2001). However, low propionic acid concentrations at later storage lengths in LBLD silages might be due to delayed involvement of *L. diolivorans* DSM 32074 in heterofermentative activity or the production of 1-propanol by *L. diolivorans* DSM 32074 instead of utilizing the pathway producing propionic acid (Zhang et al., 2010; Zielińska et al., 2017). For example, the rapid decline of pH in LBLD sweet sorghum in experiment 2 may be attributed to the activity of *L. plantarum* DSM 21762, *L. buchneri* DSM 12856, and *L. diolivorans* DSM 32074in the presence of high WSC concentrations. Furthermore, it has also been reported that *L. diolivorans* can produce acetic acid, 1,2-PD and ethanol in petri dishes containing glucose and 1,2-PD (Schein et al., 2018). Hence, it may be hard to discern in these experiments whether *L. diolivorans* DSM 32074 was engaged in the production of acetic acid or more focused on homofermentative activity. Experiment 1 demonstrates *L. diolivorans* DSM 32074 was involved in heterofermentative activity because of the increase in 1-propanol, but the low 1-propanol concentrations suggest a lower overall activity using this pathway. Interestingly, in the presence of glucose, Schein et al. (2018) did not observe propionic acid production by *L. diolivorans*, suggesting silo conditions may encourage homofermentative activity or heterofermentative activity that yields acetic acid, 1,2-PD, and 1-propanol. These same authors observed in petri dishes studies with mannitol and 1,2-PD as potential substrates, there was propionic acid production. These findings suggest extreme low WSC concentrations may be needed for *L. diolivorans* to utilize the pathway that yields propionic acid. This supports our premise that under specific silo conditions, *L. diolivorans* DSM 32074 may favor the 1-propanol pathway or the production of acetic acid over the pathway that produces propionic acid as observed in experiment 1. Additionally, although there was an effect of inoculation and storage length, the greater propionic acid concentrations found in CON sweet sorghum in experiment 2 were likely from epiphytic microbes, such as *Clostridium propionicum* (Kung et al., 2018). Overall, further research is needed to evaluate the effect of *L. diolivorans* DSM 32074 in the silo to highlight the potential production of acetic acid or demonstrate pathway preferences between 1-propanol and propionic acid production. Conversely, differences in WSC concentrations highlight the potential for heterofermentative bacteria, such as those used in this trial, to begin heterofermentative activity at different times depending on what sorghum variety is used.

While the onset of heterofermentative activity by *L. diolivorans* DSM 32074 or *L. buchneri* DSM 12856 cannot be isolated in these experiments, a shift from lactic to acetic acid occurred after the traditional 30 to 60 d lag reported by Muck et al. (2018). Acetic acid production in experiment 1 was greater in LBLD silage and increased with storage length, confirming there was *L. buchneri* DSM 12856 activity beginning at approximately 28 d (Oude Elferink et al., 2001). In experiment 2, all varieties were similarly affected by microbial inoculant and storage. However, in forage sorghum and sudangrass, CON silages had greater levels of acetic acid after 14 d of storage. This increase in acetic acid concentration without a corresponding increase in 1,2-PD could be indicative of fermentation by undesirable bacteria, such as enterobacteria, *Clostridia* or heterolactic bacteria (McDonald et al., 1991). In forage sorghum and sudangrass, there may have been fermentation by these undesirable microbes early in the silo for CON treatments, while the high WSC in sweet sorghum likely encouraged the rapid onset of homofermentative activity by LAB early in the silo and discouraged fermentation by undesirable bacteria for CON silage. Despite this, all varieties had a greater acetic acid concentration in LBLD after 56 d, as expected, after inoculation with *L. buchneri* (Oude Elferink et al., 2001).

Butyric acid is often considered a marker of undesirable fermentation and results from the fermentation of some bacteria such as *Clostridia* species (Kung et al., 2018). Although there was no effect on butyric acid observed in experiment 1 or sudangrass in experiment 2, there was an interaction effect in forage and sweet sorghum in experiment 2. Both varieties only had small concentrations detected in early storage lengths of CON silage and all detectable amounts were less than 1% DM. Although this indicates there may have been some undesirable fermentation, the small concentration does not suggest a clostridial fermentation and is likely the result of fermentation by small numbers of these bacteria.

In LBLD silage in experiment 1, greater concentrations of succinic acid were observed. This may be because of the fermentation of lactic and citric acids, which has been observed for *L. plantarum* (Lindgren et al., 1989) and is included in the tested inoculant. Additionally, the effect of inoculation and storage on 2,3-butanediol in experiment 1 is likely the result of its production by the LAB included in the inoculant used in this study, as different species in this genus have been shown to produce this fermentation byproduct (Alan et al., 2018).

Silages with greater aerobic stability are desirable because of its resistance to deterioration and reduced DM losses at feedout (Wilkinson and Davies, 2012). In experiment 1, inoculation with *L. plantarum* DSM 21762, *L. buchneri* DSM 12856 and *L. diolivorans* DSM 32074 increased aerobic stability with increasing storage length, likely because of the increase in acetic acid production and, to a lesser extent the numerically greater propionic acid production. Aerobic stability is increased by the production or supplementation of antifungal acids, such as acetic and propionic acids (Kung et al., 2018). The presence of antifungal acids suppresses the growth of yeasts, which assimilate lactic acid and promote an increase in pH after silage is exposed to air (Wilkinson and Davies, 2012). The increase in pH allows other undesirable microbes to reproliferate in the silage, contributing to the greater DM losses after aerobic exposure.

Sorghum-sudangrass may have supplied less WSC for microbial activity compared to other varieties, whereas forage and sweet sorghum appears to provide adequate concentrations (Reddy and Reddy, 2003) that could be used by LAB, determining the rate of decline in pH (Davies et al., 1998). However, the possibly slower decline in pH for sorghum-sudangrass and in the case of sweet sorghum, the high concentration of WSC, might have contributed to yeast growth in CON silage, even during the anaerobic period (Ruxton et al., 1975). These results suggest that WSC content of sweet sorghum and sudangrass in the current trial may be favorable to lactate-assimilating yeasts, but further research is warranted to elucidate this premise. This finding highlights that further investigation to determine an upper and lower limit of carbohydrates for upcoming varieties used for silage is needed to discourage yeast growth during ensiling. In experiment 2, LBLD silages reduced yeasts counts in sorghum-sudangrass and sweet sorghum, likely due to the observed effects of microbial inoculation on acetic acid concentration. In forage sorghum, the yeast counts appear to be lower overall compared to the other varieties, possibly because there were no problems related to WSC content as described previously in sweet sorghum and sudangrass, resulting in the lack of effect of microbial inoculant on yeast counts. A reduction in yeast counts is desirable because yeasts and molds can cause silage spoilage when exposed to oxygen (Muck et al., 2018). However, there was no effect of inoculation on yeast and no suppression of mold growth in experiment 1. Other studies performed on low DM (22 to 24%) corn and sorghum silages have reported additives containing *L. buchneri* inhibited mold growth with similar concentrations of acetic acid production (Filya, 2003). Based on the results in the current study, the lack of effect on yeast counts is not biologically obvious.

In addition, in experiment 2 microbial inoculation and storage length influenced the fermentation of forage sorghum by increasing SP with increasing storage length and decreasing SP with inoculation. Increasing SP indicates the breakdown of the protein matrix surrounding starch granules (Hoffman et al., 2011) with greater ensiling time (Ferraretto et al., 2015; Fernandes et al., 2020) such as in CON sorghum forage silage. Approximately 60% of proteolysis occurs due to bacterial enzymes in grain silages. Although the proteolytic activity of homofermentative LAB in silage is not known, heterofermentative inoculants may create conditions that favor proteolytic bacteria (Junges et al., 2017). Unexpectedly, the combination of *L. plantarum* DSM 21762, *L. buchneri* DSM 12856 and *L. diolivorans* DSM 32074 did not enhance concentrations of SP in comparison to CON silages. Moreover, minimal changes in starch content with greater storage length agree with the body of literature (Ferraretto et al., 2015; Fernandes et al., 2020). Greater ash concentration was observed in forage sorghum but is unlikely to be biologically significant. The small changes in EE concentration observed in this experiment, combined with the overall low concentration, suggest these effects are unlikely to be of biological significance. The concentration of WSC in experiment 2 was affected by interactions for both forage and sweet sorghum. In forage sorghum, the lower concentration in LBLD silage after 28 d of storage may be related to an increase in the rate of fermentation in LBLD beginning sometime after 14 d of storage. For sweet sorghum, the WSC concentration was much lower for LBLD than CON after 56 d of storage, suggesting a more substantial fermentation with greater production of acids. In experiment 1 there was a greater ash concentration in CON silage, but LBLD silages were only 0.9%-units lower. Therefore, the difference in ash concentration is again unlikely to be biologically significant or affect diet formulation.

Consequently, inoculation with LBLD led to a greater NDF content in sudangrass after 56 d of storage, which is known to be less digestible (Cherney et al., 1986). Besides these differences in carbohydrates, previous reports have shown similar NDF content with greater storage length than used in the present study (Sanderson, 1993; Der Bedrosian et al., 2012; Ferraretto et al., 2015), suggesting that heterofermentative inoculation’s effect on fiber portion probably decreases with greater storage length. However, the mechanism behind the increase in NDF concentration with storage length in sweet sorghum may be related to the reduction in WSC content with storage length, increasing the relative proportion of fiber in the silo.

In experiment 1, the decrease in DM content with inoculation and storage length may be partially caused by *L. diolivorans* DSM 32074. It has been reported that water is produced as a byproduct of the degradation of 1,2-PD to 1-propanol or propionic acid (Zhang et al., 2010). Therefore, the numerical increase of propionic acid with inoculation and increasing storage length, along with the increase in 1-propanol concentration, probably contributed to water production in LBLD silage from the current study.

Results of this study indicate inoculating sorghum silage with a combination of *Lactobacillus plantarum* DSM 21762, *L. buchneri* DSM 12856, and *L. diolivorans* DSM 32074 improves heterofermentative co-fermentation that allows for the accumulation of acetic acid concentration, increasing antifungal capacities of sorghum silage and thereby, its aerobic stability. Kung et al. (2018) indicated propionic acid concentrations greater than 0.3% DM is usually found in clostridial fermentations, likely a result of *Clostridium propionicum*. In the current study, high concentrations of propionic acid associated with short-term ensiling may have being from *L. diolivorans* DSM 32074 activity, or Clostridia might have outcompeted *L. diolivorans* DSM 32074 for substrate. Further research is warranted to elucidate these findings. Future research should focus on sequencing technologies to elucidate the preferential pathway for *L. diolivorans* to accelerate growth capacity in silage throughout the use of intermediates produced by *L. buchneri*.

## Data Availability Statement

The raw data supporting the conclusions of this article will be made available by the authors upon request, without undue reservation.

## Ethics Statement

The animal study was reviewed and approved by University of Florida, Institute of Food and Agricultural Sciences, Animal Care Research Committee.

## Author Contributions

LF contributed to conception and design of the study, as well as funding acquisition. ED and MP conducted the experiments, conducted the statistical analysis, and drafted the initial version of this manuscript. LG, JG, CH, CM, and MW participated in the experiments and editing of the manuscript. All authors contributed to manuscript revisions, read, and approved the submitted manuscript version.

## Conflict of Interest

The authors declare that this study received partial funding from Provita Supplements Inc. (Mendota Heights, MN, United States). The funder was not involved in the study design, collection, analysis, interpretation of data, the writing of this article, or the decision to submit it for publication. The reviewer CA declared a shared affiliation with one of the authors, JG, to the handling editor at the time of the review.
